# Institutional transparency improves public perception of lab animal technicians and support for animal research

**DOI:** 10.1371/journal.pone.0193262

**Published:** 2018-02-21

**Authors:** Katelyn E. Mills, Zetta Han, Jesse Robbins, Daniel M. Weary

**Affiliations:** Animal Welfare Program, Faculty of Food and Land Systems, University of British Columbia, Vancouver, Canada; Universidade do Porto Instituto de Biologia Molecular e Celular, PORTUGAL

## Abstract

The use of animals in research is controversial and often takes place under a veil of secrecy. Lab animal technicians responsible for the care of animals at research institutions are sometimes described as performing ‘dirty work’ (i.e. professions that are viewed as morally tainted), and may be stigmatized by negative perceptions of their job. This study assessed if transparency affects public perceptions of lab animal technicians and support for animal research. Participants (n = 550) were randomly assigned to one of six scenarios (using a 3x2 design) that described identical research varying only the transparency of the facility (low, high) and the species used (mice, dogs, cows). Participants provided Likert-type and open-ended responses to questions about the personal characteristics (warmth, competence) of a hypothetical lab technician ‘Cathy’ and their support for the described research. Quantitative analysis showed participants in the low-transparency condition perceived Cathy to be less warm and were less supportive of the research regardless of animal species. Qualitative responses varied greatly, with some participants expressing support for both Cathy and the research. These results suggest that increasing transparency in lab animal institutions could result in a more positive perception of lab animal researchers and the work that they do.

## 1. Introduction

The use of animals in research is controversial. The people who work in these institutions, including lab animal technicians responsible for the care of animals are sometimes described as performing ‘dirty work’ (i.e. professions that are viewed as morally tainted) [1], and may be stigmatized by the negative perceptions of their job [2]. Arluke (1991) conducted focus groups with technicians, researchers and other employees within 12 biomedical institutions and reported that lab technicians felt that they were perceived more negatively than researchers or students and felt that the public perceived them as the “enemy” or “murderers” [3]. Some technicians reported personal experiences of public censure and mentioned that they felt it was ‘risky’ to disclose their profession [3,4]. Although this research reveals that laboratory animal technicians perceive themselves as stigmatized, to our knowledge no work has directly assessed public views of these individuals and the job that they do.

In psychological stereotype research, there has been work to address two main personality dimensions: warmth and competence [5–7]. These two constructs are thought to explain most of the variation in judgments of other individuals and social groups [8]. We judge if someone is likable (warm) and if they are respectable (competent) [9], with warmth being more quickly recognized [10]. Previous research has examined stereotypes of workers in other professions. For example, education workers are generally considered warmer than lawyers, who are conversely viewed as more competent than the former [11]. People that work in professions caring for animals are typically viewed as warm and compassionate [12]. Technicians that work in a laboratory setting often do so because of their love of animals [13,14], suggesting that perceptions of lab animal workers might also be positive. Along with warmth and competence, social distance measures are used to assess prejudice [15] or to assess whether you prefer greater social distance from those that do not align morally or attitudinally with yourself [16]. Taken together with warmth and competence, these three measures can help us to understand a person’s social perception of another.

In recent years there have been calls for increased transparency in animal research [17]. For example, some animal research institutions have begun releasing information on the number and type of animals used in research. The Canadian Council of Animal Care (CCAC) began releasing the number of animals used in research publicly in 1996 [18]. Similar initiatives exist in both the European Union (EU) and the United States (US) [19]. However, likely in response to controversy around the use of animals in research, specifically the belief that the public is against animal research [20] but largely uninformed [4,20], some facilities have attempted to shield their activities from public view.

At our university, the largest rodent facility is in the basement of the Centre for Disease Modelling. The building signage provides no indication that animals are being used, the animal facility itself is not sign posted, and public access to view the animals and information about the research taking place is not provided. Other facilities are more open about the work and the animals used. For example, our university’s Dairy Education and Research Centre is signposted from the main highway, is open to the public, experimental animals and research protocols are available to view, and researchers and animal care staff are on hand to answer questions. The facility actively solicits public input via open houses, tour groups and school visits. Using these two examples of institutional transparency, our study had the following aims: 1) to assess whether transparency affects public perception of lab animal technicians and 2) to assess whether transparency affects public support for animal research. To achieve the first aim, we used validated scales to assess warmth and competence [8]. We also examined the qualitative responses of participants to better understand why respondents held these perceptions. We contrasted the practices used at the two facilities described above using different scenarios presented to the participants. Because these two specific facilities use different species of animals, and because attitudes towards research animal use vary with the species used [21], we also experimentally varied the study species presented in our scenarios. In addition to the two species used in these two facilities (i.e. mice and cattle) we also included a dog scenario as previous work has shown that the public often express a high level of concern regarding research on companion animals [21]. Thus an additional aim of our study was to test the effect of these species differences and any interaction between species and facility transparency on attitudes towards technicians and the research they conduct.

## 2. Methods and materials

This research was approved by the University of British Columbia’s Behavioural Research Ethics Board Protocol (H16-01852). Only participants that provided written consent were able to access the online survey. The survey instrument was created using Qualtrics (Provo, UT) online survey platform.

### 2.1 Recruitment

Participants were recruited using Amazon’s Mechanical Turk (MTurk). MTurk has been shown to result in samples that are more attentive [22] and diverse [23,24] than traditional subject pools. The survey ran from August 26-September 1, 2016. A total of 550 participants (474 US residents and 76 Canadian) were recruited. Mean age was 36 years (range 18–75); 322 (58.5%) were women.

### 2.2 Design

We used a convergent parallel mixed methods design [25] in which quantitative questions were followed by qualitative responses from participants.

Participants were told that they were participating in a study to assess whether personality traits could be accurately predicted based upon career choice [26]. Participants were then told they would be reading about a real person. Participants were then given a description of a hypothetical laboratory animal technician (“Cathy”) and a description of the facility in which she works. Participants were randomly assigned to one of six treatments using a 3x2 design (varying transparency of the facility, i.e. low or high, and species used in the described research, i.e. mice, dogs or cows). Scenarios were based on a study by Henry and Pulcino (2009) [21] in which rheumatoid arthritis is described to assess differences in study specific characteristics on support for research.

The scenarios were presented to participants as follows, with the words in brackets randomized for participants:

Cathy is a lab animal technician. She works in a facility that is testing vaccines for Rheumatoid Arthritis. Rheumatoid Arthritis is a disease that causes inflammation of the joints. A vaccine has been developed that may help with this disease. As part of Cathy's job, she artificially induces swelling in the joints of [cows, mice, dogs] to test this vaccine.

The work at this facility is seen by some as controversial, so changes have been made to [increase, decrease] interactions with the community. The research facility’s sign [is, is not] posted on the outside of the location. The name of the facility [does, does not] make it clear that animals are being used in research. Visits by the public [are, are not] allowed. Details about how the animals are housed [is, is not] publicly available.

After reading the scenario participants responded to a series of questions assessing social perception (i.e. warmth, competence, social distance) as well as questions assessing support for this research, general attitudes towards animal research and demographic questions (Table 1). Additionally, participants were asked “how transparent is this facility?” This question was included to ensure that the transparency manipulation provided discriminant validity [27]. Participants answered all social perception and support for research questions on a 7-point Likert-type scale (1 = strongly disagree,4 = neutral, 7 = strongly agree). Additionally, for warmth (3 questions) and competence (3 questions) measures, quantitative responses were followed by an open-ended question (“Please explain your answer”). An information manipulation check (IMC) question was also included to decrease noise in the data by removing participants that were not attentive [28] (see Table 1). At the end of the survey the participants were told the true intent of the study and paid $0.50.

**Table 1:** Measures used to assess public perception of lab animal technicians based on transparency of the institution.

General Construct	Question(s) Used to Assess	Adapted from
**Warmth**	How [warm, trustworthy, honest] is Cathy?	Ashton-James et al. (2014) [29]
**Competence**	How [intelligent, competent, confident] is Cathy?	Fiske et al (2002) [30]
**Social Distance**	I would be happy to have Cathy as a [neighbour, roommate, friend].	Skitka et al. (2005) [26]
**Support for this research**	I would support this facility’s use of these animals for this research.	Henry and Pulcino (2009) [19]
	This facility should not be allowed to use these animals for this research.	
	In this situation, the use of these animals for research purposes is appropriate.	
	The idea of these animals being used for this research causes me discomfort.	
**Information Manipulation Check (IMC)**	Cathy works as an animal caregiver?	

### 2.3 Analysis

Quantitative data were analyzed using the Statistical Analysis System (SAS) (version 9.4, SAS Institute Inc.). Negatively worded items were reverse scored. Internal validity was calculated using Cronbach’s alpha. Internal validity was high for all scales: 0.78 for warmth (3 measures), 0.70 for competence (3 measures), 0.93 for social distance (3 measures), 0.92 for support for this research (4 measures). On this basis we present results for the combined constructs (not the individual questions) below [31].

General linear models were constructed for each dependent measure (Warmth, Competence, Social Distance, Support for Research) testing the effect of transparency, species, the interaction between transparency and species, demographics (age, gender, level of education and political views), and the interaction between each of these demographic variables and transparency.

To analyze the qualitative responses, open-coding was used. Responses were first read and themes were developed based on participant responses; ‘transparency’ was used as an a priori theme in this framework and was not exclusive to the treatment itself. Two researchers (Mills and Han) independently coded a sub-sample to determine consistency in the themes and sub-themes. When there was disagreement between researchers this was discussed until a consensus could be reached. The finalized codes were then used by one researcher (Han) to code all of the responses, including the ones initially coded. Qualitative responses that did not answer the question (ie. “Please explain your answer.”) were excluded [32]. If multiple themes were present in individual participant responses, all themes were recorded. If a theme was present in more than one place in any given response it was only coded once. The focus of the analysis was not to determine the prevalence of themes but instead to show the range of responses from participants.

When coding the qualitative responses from participants several themes began to emerge and were consistent across all character traits (Warm, Honest, Trustworthy, Competent, Confident, Intelligent). Additionally, the treatment affected the positive or negative reaction to the theme, but the themes remained constant. Therefore, the qualitative results will be presented by theme as opposed to by treatment.

## 3. Results

A total of 614 participants were recruited for this study. After excluding incomplete surveys (n = 8), failed attention check (n = 44) [28], and invariant responses (n = 12) [33] our final sample consisted of 550 participants. Fisher’s exact tests of independence revealed that treatment demographics did not differ among the six treatments (see Table 2).

**Table 2:** The number (and percentage) of participants in each demographic category, and Fisher’s exact test assessing any contingency with treatment (LT = Low Transparency; HT = High Transparency).

Demographic	Total	LT Dog	LT Cow	LT Mouse	HT Dog	HT Cow	HT Mouse	*p*
**Total Participants**	550 (100)	93 (16.91)	95 (17.27)	91 (16.55)	83 (15.09)	90 (16.36)	98 (17.82)	
**Age**								0.864
18–24	70 (12.73)	10 (1.82)	16 (2.91)	12 (2.18)	11 (2.00)	13 (2.36)	8 (1.45)	
25–34	244 (44.36)	43 (7.82)	39 (7.09)	41 (7.45)	35 (6.36)	41 (7.45)	45 (8.18)	
35–44	107 (19.45)	14 (2.55)	21 (3.82)	20 (3.64)	13 (2.36)	19 (3.45)	20 (3.64)	
45–54	66 (12.00)	13 (2.36)	13 (2.36)	9 (1.64)	14 (2.55)	6 (1.09)	11 (2.00)	
55–64	52 (9.45)	11 (2.00)	6 (1.09)	6 (1.09)	8 (1.45)	10 (1.82)	11 (2.00)	
65 or above	11 (2.00)	2 (0.36)	0 (0.00)	3 (0.55)	2 (0.36)	1 (0.18)	3 (0.55)	
**Gender**								0.658
Female	322 (58.5)	54 (58.1)	55 (57.9)	55 (60.4)	42 (50.6)	57 (63.3)	59 (60.2)	
Male	228 (41.5)	39 (41.9)	40 (42.1)	36 (39.6)	41 (49.4)	33 (36.7)	39 (39.8)	
**Education**								0.293
Did not graduate high school	3 (0.55)	1 (0.18)	2 (0.36)	0 (0.00)	0 (0.00)	0 (0.00)	0 (0.00)	
High school graduate	39 (7.09)	7 (1.27)	12 (2.18)	4 (0.73)	3 (0.55)	6 (1.09)	7 (1.27)	
Some college, no degree	139 (25.27)	27 (4.91)	15 (2.73)	20 (3.64)	21 (3.82)	26 (4.73)	30 (5.45)	
Associate/Trade degree	75 (13.64)	12 (2.18)	10 (1.82)	19 (3.45)	9 (1.64)	13 (2.36)	12 (2.18)	
Bachelor’s degree	208 (37.82)	28 (5.09)	42 (7.64)	38 (6.91)	36 (6.55)	32 (5.82)	32 (5.82)	
Graduate degree	84 (15.27)	17 (3.09)	14 (2.55)	10 (1.82)	14 (2.55)	13 (2.36)	16 (2.91)	
No answer	2 (0.36)	1 (0.18)	0 (0.00)	0 (0.00)	0 (0.00)	0 (0.00)	1 (0.18)	
**Political views**								0.246
Conservative	129 (23.45)	15 (2.73)	24 (4.36)	21 (3.82)	22 (4.00)	19 (3.45)	28 (5.09)	
Moderate	170 (30.91)	36 (6.55)	23 (4.18)	25 (4.55)	23 (4.18)	27 (4.91)	36 (6.55)	
Liberal	251 (45.64)	42 (7.64)	48 (8.73)	45 (8.18)	38 (6.91)	44 (8.00)	34 (6.18)	
**Household income**								0.086
$0- $49,999	267 (48.55)	47 (8.55)	53 (9.64)	47 (8.55)	32 (5.82)	42 (7.64)	46 (8.36)	
$50,000 - $99,999	219 (39.82)	39 (7.09)	34 (6.18)	35 (6.36)	38 (6.91)	41 (7.45)	32 (5.82)	
$100,000 +	64 (11.64)	7 (1.27)	8 (1.45)	9 (1.64)	13 (2.36)	7 (1.27)	20 (3.64)	

### 3.1 Quantitative results

The transparency check was successful; participants assigned to the high transparency condition scored transparency higher than did those in the less transparent condition (5.1±1.51 vs. 2.3±1.56; F_1,548_ = 474.0, P = 0.0001).

**Fig 1:**
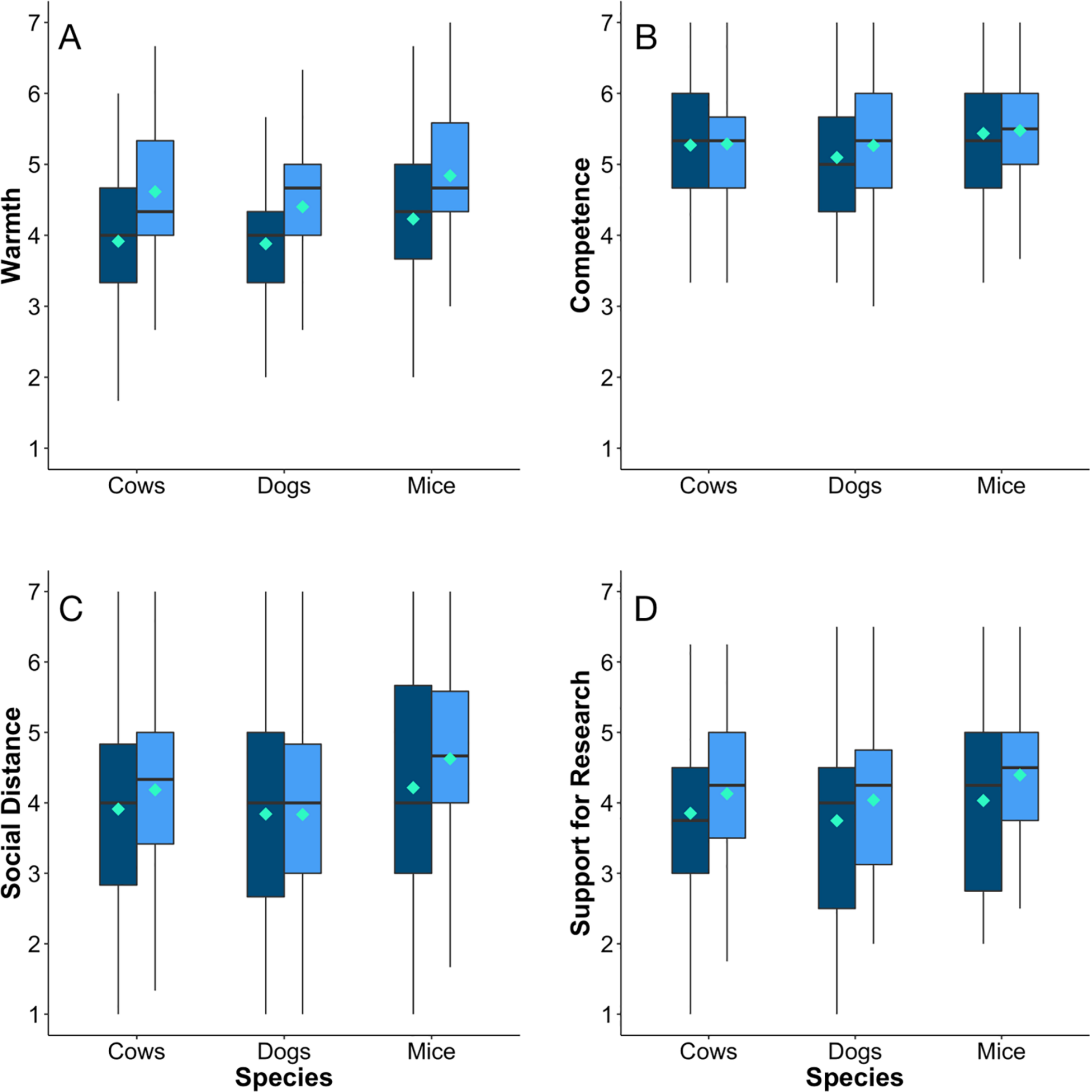
Responses by participants asked questions designed to assess a) warmth, b) competence, and c) social distance for the hypothetical lab animal technician “Cathy,” as well as d) support for the research she does. Participants were randomly asssigned to scenarios that described Cathy as working at either a low (dark blue) or high transparency (light blue) institution, using three different species of research animal (cows, dogs and mice). All individual questions were asked on a 7 point Likert-type scale (1 = not at all; 4 = neutral, 7 = extremely); responses for each of the composite measures shown below were averaged across individual questions. The box plots show the mean (central square), median (solid horizonal line in the middle of the box), 25^th^ and 75^th^ percentiles (the upper and lower limits of the box) and 10^th^ and 90^th^ percentiles (verticle lines extenting above and below the box).

The lab animal technician “Cathy” was perceived as warmer in the high transparency compared to the low transparency condition (Fig 1; 4.6±0.06 vs. 4.0±0.06; F_1,534_ = 48.9, P<0.0001), and tended to be perceived as more socially distant in the low (4.2±0.06) versus high transparency treatment (3.9±0.06; F_1,534_ = 3.6, P = 0.06; see Table 3). Perceived competence did not vary with transparency (F_1,534_ = 1.2, P = 0.28), but participants were more supportive of research in the high transparency versus low transparency treatment (4.2±0.06 vs. 3.9±0.06; F_1,534_ = 12.4, P = 0.0005).

**Table 3:** Effect of participant demographics (gender, political leaning, age, education, and income) on composite responses to questions designed to assess a) warmth, b) competence, and c) social distance for the hypotheitical lab animal technician “Cathy,” as well as d) support for the research she does. All individual questions were asked on a 7 point Likert scale (1 = not at all; 7 = extremely); responses for each of the composite measures below were averaged across individual questions. The effects of participant demographics on these responses are shown by coefficients (slope (B) and S.E.), and the t-value and corresponding probability that these values differ from 0. These values are from general linear model that also included the main effects of transparency and species, and the relevant 2-way interactions. Bold values indicate *p* = < .05.

	Warmth				Competence				Social Distance				Support for Research			
Variable	Type 1 SS	Mean Square	F value	*p*	Type 1 SS	Mean Square	F value	*p*	Type 1 SS	Mean Square	F value	*p*	Type 1 SS	Mean Square	F value	*p*
Transparency	**53.498**	**53.498**	**48.34**	**< .0001**	0.945	0.945	1.16	0.283	**8.550**	**8.550**	**3.55**	**0.060**	**14.253**	**14.253**	**12.37**	**0.0005**
Species	**6.955**	**6.955**	**6.28**	**0.013**	2.903	2.903	3.56	0.060	**13.383**	**13.383**	**5.56**	**0.0187**	**4.702**	**4.702**	**4.08**	**0.044**
Female (*vs male*)	1.982	1.982	1.79	0.181	0.0002	0.0002	0.00	0.987	**39.902**	**39.902**	**16.58**	**< .0001**	**18.112**	**18.112**	**15.72**	**< .0001**
Liberal (*vs conservative*)	0.357	0.357	0.32	0.570	0.010	0.010	0.01	0.911	2.428	2.428	1.01	0.316	0.002	0.002	0.00	0.969
Age	2.698	2.698	2.44	0.119	**4.175**	**4.175**	**5.12**	**0.024**	**15.615**	**15.615**	**6.49**	**0.011**	3.802	3.802	3.30	0.070
Education	0.051	0.051	0.05	0.830	**4.240**	**4.240**	**5.19**	**0.023**	1.271	1.271	0.53	0.468	3.978	3.978	3.45	0.064
Income	1.044	1.044	0.94	0.332	0.042	0.042	0.05	0.820	3.012	3.012	1.25	0.264	**8.215**	**8.215**	**7.13**	**0.008**

Participant responses also varied in relation to the species used in the scenario (see Fig 1); Cathy was perceived to be more warm when the scenario used mice (4.5±0.08) and cows (4.3±0.08) than when using dogs (4.1±0.08; F_2,534_ = 6.7, P = 0.001). Similar variation in response to species was found for competence (F_2,534_ = 4.4, P = 0.01), social distance (F_2,534_ = 6.7, P = 0.001), and support for research (F_2,534_ = 4.4, P = 0.01). The interaction between species and transparency was not significant for any of the four constructs.

There were no main effects of participant demographics on perceptions of warmth (P>0.1 in all cases). However, we did find an interaction between participant age and transparency for this measure (F_1,534_ = 5.2, P = 0.02). This interaction was driven by older participants in the high transparency condition reporting reduced perceptions of warmth.

Age and education of participants both affected perceived competence (F_1,534_ = 5.1, P = 0.02 and F_1,534_ = 5.2, P = 0.02, respectively); older and more educated participants perceived Cathy as less competent. There were no interactions between the demographic effects and transparency for this measure.

Perceived social distance was affected by participant gender and age (F_1,534_ = 16.6, P<0.0001 and F_1,534_ = 6.5, P = 0.01, respectively); participants who were female and younger reported less social distance between themselves and Cathy. There was an interaction between transparency and income (F_1,534_ = 7.5, P = 0.006), driven by participants with higher incomes having decreased social distance, but only in the high transparency condition.

Support for research was affected by gender and income (F_1,534_ = 15.7, P<0.0001 and F_1,534_ = 7.1, P = 0.008, respectively); females and those with lower incomes were less likely to support the research. Participants who were younger and more educated tended to be more supportive of the research (F_1,534_ = 3.3, P<0.07 and F_1,534_ = 3.5, P = 0.06, respectively). There were no interactions between demographics and transparency for this measure.

### 3.2 Qualitative results

#### 3.2.1 Personal attributes

Participants commented on Cathy’s character, both positively and negatively (see Table 4). Positive comments from respondents described Cathy as “humble”, “compassionate”, “strong”, and “caring”. In the words of one participant, “this seems like a noble profession, something that someone with a caring heart would be involved in” (P106). Alternatively, when describing Cathy negatively participants used words such as “lack of compassion”, “secretive”, “unethical”, and “detached”. In one participant’s words “it seems that Cathy would have to be a fairly shady, unethical person to work at such a place” (P366). Personal attributes associated with Cathy were due to the work that she did and the institution where she worked. In the words of one participant:

“She's warm in the sense that she is doing a job that has the potential to help people which shows she is caring and compassionate. But that same job has her hurting the mice (or what I consider to be hurting) so that shows the opposite of warm: cold and not compassionate.” (P289)

**Table 4:** Themes coded for qualitative responses to perception of lab animal technicians in high and low transparency institutions.

Theme	Description
Transparency	Reference to public openness, transparency or interaction with the public; reference to the company making decisions, not Cathy
Nature of the Research	Reference to species used, procedures performed, validity of the research
Job Perception	Reference to what a lab animal technician does or what is required of them; what is believed to be in control of the employee.
Personal Attributes	Reference to character attributes of Cathy (i.e. detached, helpful, unethical)
Not Enough Information	Reference to not enough information or belief that link cannot be made between personality and job description; intuition or “gut feeling”.

#### 3.2.2 Job perception

Participants described specific job traits including Cathy’s job qualifications, the responsibilities of her position and loyalty to the institution that she works for. When asked about Cathy’s competence (competent, intelligent, confident) participants were more likely to give reasons related to Cathy’s qualifications (e.g. “she works in the science field, so she has to be somewhat intelligent” P144) and the responsibilities in her position (e.g. “the company's success depends of Cathy's ability to perform her job with extreme care” P354). Additionally, there were respondents that felt this was just a job for Cathy (e.g. “Cathy is just a lab technician and sometimes you need to do what you need to do to make a living” P522).

#### 3.2.3 Transparency

Transparency was a prominent theme in regards to questions concerning Cathy’s warmth (honesty, trustworthiness, warmth). For example, one participant (in the more transparent treatment) commented that “openly disclos[ing] the kind of work they do, open[ing] their facilities for tours and provid[ing] information to the public” (P41) reflected positively on Cathy. In contrast, a participant in the low transparency condition commented:

“The fact that she works with animals makes me believe she has empathy and compassion, but this is offset by the fact that she is willing to hide her activities in order to avoid public scrutiny and disapproval. This tells me she is more committed to her job and her research than she is to ethical treatment of animals.” (P54)

Participants perceived Cathy to be trustworthy because she was working in a low transparency environment. For example, one participant said that “if it is a secretive place she is working then she must be somewhat trustworthy” (P20). It could be argued that this view conflates trust and loyalty, and suggests that future work should specifically consider loyalty measures.

Participants felt the responsibility for transparency was Cathy’s as opposed to that of the institution. For example, when asked about Cathy’s confidence one participant responded that:

“Cathy must be confident in her abilities to allow community involvement in her testing. If she were not confident, she would be reluctant to allow such public scrutiny” (P52). Others believed that the responsibility of transparency rested with the research institution. A third set of respondents described that while the institution set the policy around transparency, Cathy was complicit by working there. In the words of one participant: “the company she works for is being shady about the kind of work they are doing, therefore she falls under the umbrella of shade” (P287).

#### 3.2.4 Cannot make a judgment

Not surprisingly, participants felt that they could not make a judgment on Cathy based only on the limited information we provided. For example, “I don't have enough information to determine if she is a cold or warm person. She has a job that has a lot of controversy surrounding it, but I can see both sides of the argument. I can't judge her one way or the other” (P293).

Perhaps for this reason, some participants described that their response was a “gut feeling” or an “assumption”. In addition, participants felt that they could not make the link between personality and profession, with one participant stating that “one's job does not completely describe their personality” (P61).

#### 3.2.5 Nature of the research

‘Nature of the research’ was a theme that appeared in a range of responses. This was partly due to the different species (cows, mice, dogs) presented in the scenario. For example, one participant wrote “anyone who would harm dogs cannot have a warm heart” (P261). Interestingly, one participant felt that using larger mammals inspired a higher level of confidence in Cathy (e.g. “she's pretty confident in her research enough to use cows and not mice” P292). Participants made positive comments within this theme. For example, a participant (P71) commented that “I think [Cathy] has very good intentions because she wants to help people. It might seem as cruel to test on animals but that does not mean she is uncaring. I think she must be strong to actually bottle her feelings up in order to do her work so it might not be as obvious how warm she might be”.

Participants responded with reasons relating to what was being done to the animals. For example, one participant described that “while the fact that Cathy induces inflammation in cow's joints may make her seem like a bad person, it is her job” (P35). Similarly, one participant drew on personal experience when relating to the animals being tested on. This participant stated:

“While I understand that this is her job, and ultimately the end result is to help others, I do not believe she is a very warm person if she has no problem inducing dogs with RA. I am a current sufferer of RA, and I wouldn't want to give this to any human or animal on purpose” (P101).

Participants expressed ambivalence associated with the good and the bad in Cathy’s work. For example, one person (P58) wrote “She is caring because she wants to help treat Rheumatoid arthritis but at the same time she is testing on animals”.

## 4. Discussion

### 4.1 Quantitative results

Participants perceived Cathy as less warm when she worked in the less transparent institution, but transparency did not affect perceived competence or social distance. It should be noted that the participants in this sample generally did not have extreme perceptions of Cathy. In both the high and low transparency treatments quantitative responses were largely neutral, approximately 4, on the Likert-type scale. This result contrasts with the perception within lab animal institutions that the public is highly negative towards people who use animals in research [3,4].

Our results also show that increased transparency results in increased support for the research. This is an important result as there are numerous initiatives to increase transparency in animal research institutions [15]. For example, the Montreal Declaration [34] and Basel Declaration [35] have been introduced with the aim to increase transparency. Public support for research is highly dependent on factors such as economics, geography, politics, among others [36]; the results of the current study show that transparency is also important for public support.

The results of the current study also show an effect of species. Other studies have also found that species influences support for research; research that involves dogs is more opposed than that on mice [19], a result that was also seen in this study. Importantly, we found no interaction between transparency and species, suggesting that increased transparency increases public support for research, and perceptions of lab employees, irrespective of species used.

Demographic factors, including age, income, gender and education, affected participant responses, but there were very few interactions between demographics and transparency indicating that the positive effects of increased transparency are consistent across these features. As in the current study, previous work has shown that female participants are less likely to support research [19, 37]. In previous studies, age has had variable effects on support for research, with some studies showing positive correlations and others negative [37]. This inconsistency was also shown in this study, in that younger participants were more supportive of research but also believed Cathy to be less competent. Increased education has a positive correlation with support for research [36], a result also shown in this study. It is unclear why increased education resulted in less perceived competence of Cathy, and why increased income affected both social distance and support for research. This could be a limitation of how we considered education as a demographic measure (as level of educational attainment), and not taking into account specialization. Future work may wish to find out about education in biomedical sciences that is more likely to expose students to animal use. Similarly, political affiliation did not affect the results. Political options given to participants did not reflect the names of political parties in these two countries but instead represent a range of political affiliations.

### 4.2 Qualitative results

The qualitative responses were varied in both themes that emerged and the positive or negative valences to those themes. As expected, transparency was a theme that many participants discussed. Of interest also is the variety of responses that participants gave, including perceptions of the job itself, the research and personal attributes they ascribed to Cathy.

Qualitative responses showed that at least some participants felt that it was unfair to scapegoat Cathy for work and policies set by the institution, even though our quantitative results showed that participants were swayed by this factor. In their qualitative comments, some participants criticized Cathy for not disclosing information to the public and others praised her for opening the doors and allowing people in. In many institutions, lab technicians do not have much control over policies. Also, Holmberg and Ideland (2010) found that lab technicians did not believe it was their job to persuade skeptical members of the public to accept their research [18]. While this was not specified in this study, many participants assumed that Cathy was in control of the transparency of this hypothetical institution. This could be applied to blame-shifting, in which the mere presence of an intermediary (in this case, Cathy) can shift the blame from the culpable party even if that intermediary is incapable of producing a fair outcome [38]. We encourage future research to specifically examine blame shifting in this context.

### 4.3 General discussion

Previous research has assessed public perceptions of individuals working in different professions [39], but to our knowledge this is the first study that assessed attitudes towards animal researchers. A common perception within the lab animal community is that the public has an overwhelmingly negative view of this activity [3], but the current results show considerable variation in attitudes, including some very negative but also some positive and more ambivalent perspectives.

In their comments, several participants said that they could not make the link between a person’s profession and their character, but the quantitative results show differences between the low and high transparency treatments. This result suggests that people may have unconscious biases that upon reflection they consider unfair. Intuitive judgments are produced quickly [40] and it could be argued that these quick judgments are more likely to be evident in the quantitative results while the qualitative responses reflect slower, more reasoned judgments.

The results of this study show that institutional transparency can affect constructs related to employees and support for research. This suggests that increased transparency could increase acceptance of animal research, but other factors are clearly important. The current study fits in well with previous research on attitudes towards the use of animals in research that shows variation in support for research using different animal species [19]. Other factors known to influence attitudes to animal research include the invasiveness of the research and the extent to which the stated goals of the study are viewed as necessary [19]. Other work would be needed to examine potential confounds between transparency and other factors, but on the basis of the current results we conclude that animal care workers, and the research that they do, are viewed more positively when the institution provides greater transparency. Additionally, while the study focused on the public’s perception of lab animal technicians, further research could explore how people within laboratory animal research perceive others within the industry.

There are limitations to the current study, including that this study is based only on US and Canadian participants. Further work should explore other jurisdictions. Additionally, the distinction between perception of the institution and perception of the individual employee was not explored. Similarly, we did not account for contextual cues inherent in labelling the hypothetical animal care technician ‘Cathy’. This name implies gender and perhaps other attributes that were not the focus of our study. We encourage future research to consider explore a range of contextual factors to better understand the generality of the results we report here. Job responsibilities and even titles for lab technicians vary by facility, and this was not accounted for in our study. We assumed that most participants would have limited knowledge of lab animal research practices and felt that the term lab animal technician was an appropriate starting point; future work could probe respondents for differences in the level of their background understanding and knowledge, and could experimentally vary the use of different terms and job descriptions. Finally, this study assessed only two of many possible examples of transparency in animal research laboratories; future studies could examine a wider range of examples, including open access and commenting on research protocols.

## 5. Conclusion

Increasing transparency in animal research facilities can improve public perception of the workers responsible for the research and of the research conducted. This effect of increased transparency was similar for research scenarios using mice, cattle and dogs. We conclude that research organizations and the people who work within them benefit from adopting policies and practices that allow greater levels of openness to the public.
